# Associations Between Asthma Control, Insomnia Severity, and Psychosocial Outcomes: A Cross-Sectional Mediation Analysis

**DOI:** 10.3390/healthcare14111446

**Published:** 2026-05-23

**Authors:** Selda Günaydın, Meltem Hazel Şimşek, Hayriye Bektaş Aksoy, Şaban Melih Şimşek

**Affiliations:** 1 Department of Chest Diseases, Faculty of Medicine, Giresun University, 28100 Giresun, Türkiye; bektas_hayriye@hotmail.com (H.B.A.); melih.simsek@giresun.edu.tr (Ş.M.Ş.); 2 Department of Psychiatry, Faculty of Medicine, Giresun University, 28100 Giresun, Türkiye; hazel.simsek@giresun.edu.tr

**Keywords:** asthma control, insomnia, social anhedonia, functional outcomes, mediation analysis, psychosocial outcomes

## Abstract

**Background/Objectives:** Insomnia is highly prevalent among patients with asthma and has been associated with systemic inflammation, reduced lung function, and increased mortality. This study investigated whether insomnia mediates the relationship between asthma control and psychosocial dysfunction, including social anhedonia and functional impairment. **Methods:** This cross-sectional study included 153 adults with physician-diagnosed asthma classified as controlled (*n* = 51) or uncontrolled (*n* = 102) according to the Asthma Control Test (ACT). Insomnia severity was assessed using the Athens Insomnia Scale (AIS), social anhedonia using the Revised Social Anhedonia Scale (RSAS), psychological distress using DASS-21, and functional impairment using the Functioning Assessment Short Test (FAST). **Results:** Uncontrolled asthma was associated with significantly higher insomnia severity and greater depression, anxiety, and stress levels (all *p* < 0.001). Asthma control emerged as the strongest independent predictor of insomnia severity (β = −0.451, *p* < 0.001). Although asthma control was not directly associated with social anhedonia or functional impairment, insomnia significantly mediated these relationships. The indirect effect of asthma control on social anhedonia via insomnia was significant (B = −0.1162, 95% CI [−0.2384, −0.0029]), as was the indirect effect on functional impairment (B = −0.4953, 95% CI [−0.8656, −0.1038]). Spirometric indices were not independently associated with psychosocial outcomes. **Conclusions:** Insomnia may represent an important intermediary process linking poor asthma control to psychosocial dysfunction. These findings highlight the clinical importance of assessing sleep disturbances in asthma patients and suggest that insomnia may contribute to broader psychosocial impairment beyond respiratory symptoms alone.

## 1. Introduction

Asthma is one of the most prevalent chronic respiratory diseases worldwide and continues to impose a substantial public health burden across age groups. Asthma is a chronic inflammatory airway disease characterized by variable airflow limitation, bronchial hyperresponsiveness, and persistent airway inflammation. Although asthma management focuses on symptom control and reducing future risk, adequate control is not achieved in a significant proportion of patients despite optimal pharmacotherapy [1]. Increasing evidence suggests that asthma outcomes are associated not only with airway physiology but also by psychosocial and behavioral factors [2,3]. Asthma significantly impairs health-related quality of life and daily functioning. Patients frequently report limitations in occupational performance, social engagement, and physical activity, even in the absence of severe airflow obstruction [3]. Evidence suggests that uncontrolled asthma is associated with increased psychological distress, including a worsening of anxiety rates and depressive symptoms, which further exacerbate disease perception and symptom burden [4,5]. Depression, anxiety, and stress symptoms are highly prevalent in asthma populations and have been associated with poorer disease perception, impaired sleep quality, and reduced quality of life. These findings emphasize that asthma outcomes are not only determined by airway physiology but also shaped by complex interactions between respiratory, psychological, and behavioral factors [2]. Therefore, asthma control should not be conceptualized exclusively in terms of spirometric parameters; rather, it represents a multidimensional construct encompassing physiological stability, emotional well-being, and functional capacity [1].

Sleep disorders are increasingly recognized as a critical yet underappreciated dimension of asthma morbidity. Asthma patients frequently report poor sleep quality, difficulty falling asleep or staying asleep, and nighttime awakenings associated with respiratory symptoms [6]. Nocturnal asthma symptoms frequently impair sleep continuity and are associated with poorer sleep quality among asthma patients [7]. Epidemiological evidence suggests a bidirectional relationship between asthma and insomnia; insomnia increases the risk of asthma, while poorly controlled asthma exacerbates sleep disturbance [8]. Beyond the burden of nocturnal symptoms, insomnia has been associated with systemic inflammatory activation and increased sympathetic nervous system activity, mechanisms that can persist both airway inflammation and central hyperarousal [9,10]. Chronic sleep disturbance has also been associated with impaired daily functioning, reduced cognitive performance, and decreased quality of life, highlighting its wider clinical significance [10]. Overall, these findings suggest that insomnia is not only a secondary complaint in asthma but a common and clinically significant condition that may be closely related to disease control. Therefore, understanding whether insomnia acts as an intermediary mechanism linking asthma control to psychosocial dysfunction may provide clinically relevant insight beyond conventional symptom-based assessment.

Psychological comorbidity presents a critical but frequently overlooked dimension of the burden of asthma. Prospective data also suggest that depression not only co-occurs with asthma but may also increase the risk of asthma onset, highlighting the bidirectional interaction between respiratory and emotional disorders [11]. Chronic diseases, including asthma, are often associated with social withdrawal, reduced social participation, and decreased involvement in previously satisfying activities, particularly in the presence of persistent symptoms and uncertainty regarding disease control [2]. In this context, social anhedonia, defined as a diminished capacity to enjoy social interactions and reduced social motivation, has emerged as a transdiagnostic construct reflecting altered reward processing and motivational deficits [12,13]. Social anhedonia has been consistently associated with interpersonal dysfunction, occupational limitations, and broader reductions in overall functioning [12,14]. In chronic medical conditions, reduced social motivation and diminished engagement in rewarding interpersonal experiences may contribute to poorer quality of life, reduced social participation, and impaired disease self-management. Therefore, social anhedonia may represent an underrecognized dimension of psychosocial burden in asthma populations. Although extensively investigated in psychiatric and behavioral research, social anhedonia remains largely understudied in patients with asthma. Reduced social motivation and diminished engagement in rewarding interpersonal experiences may have important implications for quality of life and functional impairment beyond traditional measures of mood symptoms in chronic respiratory disease.

Functional impairment is a clinically significant outcome that extends beyond symptom severity and physiological indicators. In chronic medical conditions, functional status reflects the patient’s ability to maintain independence, occupational performance, cognitive functioning, interpersonal relationships, and participation in daily activities. In asthma, impairments in these domains may persist even in the absence of severe physiological limitation, suggesting that disease burden cannot be fully explained by respiratory parameters alone. Therefore, evaluating functional outcomes may provide clinically meaningful information beyond conventional respiratory measures. Psychological distress, sleep disturbance, and reduced social participation may independently contribute to occupational and interpersonal dysfunction in asthma populations [2]. These findings highlight the complex and multifactorial relationship between clinical symptom burden and daily functioning, emphasizing the importance of evaluating functional outcomes within an integrated biopsychosocial framework.

The literature supports relationships between asthma control and insomnia, asthma and psychological distress, and insomnia and functional impairment [3,8,11]. However, previous research has largely focused on direct relationships among asthma symptoms, mood disturbances, and sleep problems, while the potential indirect pathways linking poor asthma control to psychosocial outcomes remain insufficiently explored. In particular, few studies have investigated whether insomnia functions as a mediating mechanism between asthma control and psychosocial outcomes such as social anhedonia and functional impairment. Social anhedonia, a construct related to diminished social reward processing and motivation, remains largely understudied in asthma populations despite its potential relevance to functional decline associated with poor asthma control. Insomnia may be particularly relevant in this context because persistent sleep disturbance has been associated with impaired emotional regulation, reduced daytime functioning, diminished social engagement, and increased psychological distress across chronic medical conditions. Therefore, insomnia may represent a potential intermediary process through which poor asthma control contributes to broader psychosocial dysfunction. Therefore, examining insomnia as a potential intermediary process may provide a more integrative understanding of how physiological dysregulation translates into broader psychosocial impairment in asthma.

The aim of this study is to examine the relationships between asthma control, insomnia severity, social anhedonia, and functioning, and to evaluate the possible mediating role of insomnia in these relationships.

**H1:**  *There is a significant relationship between asthma control level and insomnia severity; poorer asthma control is associated with higher insomnia severity.*

**H2:**  *Insomnia severity is significantly associated with psychological distress, social anhedonia, and functional impairment in patients with asthma.*

**H3:**  *Insomnia severity may play a partial or complete mediating role between asthma control and social anhedonia.*

**H4:**  *Insomnia severity may play a mediating role between asthma control and functionality.*

## 2. Materials and Methods

### 2.1. Study Design and Sample

The study has a cross-sectional design. Sample size estimation was performed using G*Power software (version 3.1.9.7; Heinrich Heine University Düsseldorf). Assuming a Type I error rate of 5%, statistical power of 95%, and a medium effect size (f^2^ = 0.15) for a multiple linear regression analysis with eight independent variables, the required minimum sample size was calculated to be approximately 96 participants.

However, since mediation analyses were also planned for the study and larger samples were required to reliably identify indirect effects, the target sample size was increased. Patients were recruited consecutively from the pulmonary diseases outpatient clinic between January 2026 and March 2026 dates. The final sample consisted of 153 participants, which provides sufficient statistical power for the planned analyses. For mediation analyses, bias-corrected bootstrap procedures with 5000 resamples were applied, and indirect effects were interpreted based on 95% confidence intervals [15]. Mediation analyses were performed using the PROCESS macro for IBM SPSS Statistics (version 4.2; IBM Corporation, Armonk, NY, USA).

During the recruitment period, 186 patients diagnosed with Asthma presented to the Giresun Training and Research Hospital Chest Diseases Outpatient Clinic. Of these, 18 patients were excluded due to additional physical illnesses and comorbid chest pathologies, 12 due to inability to complete spirometry testing, and 3 due to psychiatric comorbidities that could interfere with questionnaire validity (e.g., psychotic disorders, bipolar disorder, neurocognitive disorders, or substance use disorders, excluding nicotine dependence). The remaining 153 individuals constituted the final study sample. Patients diagnosed with Asthma by a pulmonologist according to the Global Initiative for Asthma (GINA) 2025 guidelines were included as participants [1]. Disease severity was staged by the pulmonologist and classified into two groups based on asthma control status using the Asthma Control Test (ACT): controlled asthma (*n* = 51) and uncontrolled asthma (*n* = 102).

Exclusion criteria for our study include having experienced a severe acute exacerbation within the last 4 weeks, history of neurological or psychiatric disease (e.g., dementia, schizophrenia, bipolar disorder, etc.), severe hearing/vision loss or cognitive impairment that would prevent completion of the tests, having other serious chronic lung diseases (e.g., interstitial lung disease, pulmonary fibrosis, sarcoidosis), history of serious cardiovascular disease (myocardial infarction within the last 6 months, uncontrolled hypertension, advanced heart failure), having undergone major surgery within the last 3 months, those using montelukast due to psychiatric side effects, history of alcohol or substance dependence.

### 2.2. Data Collection Tools

#### 2.2.1. Sociodemographic and Clinical Data Form

This form includes basic sociodemographic information such as age, gender, and occupation. Clinical data such as Asthma control duration, pack-years of smoking, comorbidities, medications used, and the number of attacks in the past year are also recorded.

#### 2.2.2. Clinical Parameters/Objective Measurements

Asthma control was assessed using the Asthma Control Test (ACT), a validated 5-item self-report questionnaire evaluating symptom control over the past 4 weeks [16,17]. Higher scores indicate better asthma control. According to the Turkish validation study of the ACT, patients with ACT scores ≥20 were classified as having controlled asthma, whereas those with ACT scores <20 were classified as having uncontrolled asthma [17]. Pulmonary function tests (FEV_1_ and FVC % predicted) were measured using standardized spirometry procedures.

#### 2.2.3. Assessment of Insomnia

Insomnia severity was evaluated using the Athens Insomnia Scale (AIS), an 8-item self-report measure assessing sleep difficulty and daytime dysfunction. Higher total scores reflect greater insomnia severity [18,19].

#### 2.2.4. Depression, Anxiety, and Stress Scale—21 (DASS 21)

This scale was used to measure depression, anxiety, and stress levels in the sample. This scale was developed by Henry and Crawford (2005) [20]. The scale has a 4-point Likert-type response format. It consists of three subscales: depression, anxiety, and stress. These subscales consist of seven items, and the scores are summed and multiplied by two. Higher scores reflect a more negative mood. A Turkish version was also produced, and the internal consistency coefficients of the Turkish version were reported as 0.87 for the depression subscale, 0.85 for anxiety, and 0.81 for stress [21].

#### 2.2.5. Functioning Assessment Short Test (FAST)

This scale was developed by Rosa et al. to assess functioning [22]. It consists of 24 items on a four-point Likert scale scored from 0 to 3. The scale assesses functionality across six dimensions: autonomy, occupational functionality, cognitive functionality, financial status, interpersonal relationships, and leisure activities. In addition, the total score obtained from all items is used to determine the overall level of functionality. On the scale, where the total score ranges from 0 to 72, higher scores indicate greater functional impairment. The Turkish validity and reliability study of the scale was conducted by Aydemir et al. [23]. The Cronbach’s alpha coefficient was reported as 0.96.

#### 2.2.6. Social Anhedonia Scale (RSAS)

The RSAS was used to assess participants’ level of enjoyment derived from social interactions and interpersonal relationships. The scale was developed by Eckblad et al. (1982) [24]. This self-report scale consists of 40 items, and responses are given as “Yes” or “No.” High scores on the scale indicate that the individual’s enjoyment of social interactions has decreased, meaning that their level of social anhedonia is high. The Turkish version of the RSAS has demonstrated satisfactory psychometric properties. The Turkish validity and reliability study was conducted by Cihan et al. (2015) [25]. The study reported that the factor structure of the scale was consistent with the original form, the internal consistency coefficient (Cronbach’s alpha) was 0.86, and the test–retest reliability was 0.82.

### 2.3. Ethical Approval

This study was approved by the Giresun Training and Research Hospital Ethics Committee [Decision No: (09); Date: 7 January 2026]. Written informed consent was obtained from all participants. The study was conducted in accordance with the principles of the 2013 Declaration of Helsinki.

### 2.4. Statistical Analysis

The data obtained from the study were statistically analyzed using the Statistical Package for the Social Sciences (SPSS) Statistics for Windows^®^, version 21.0, and SPSS Amos 21.0.0 (IBM Corp., Armonk, NY, USA) software. Descriptive statistics were presented as frequency and percentage distributions, median (interquartile range), mean, and standard deviation values. The normality of numerical variables was assessed, and comparisons were made accordingly. Student’s *t*-test and ANOVA were used for variables showing a normal distribution, while the Mann–Whitney U and Kruskal–Wallis tests were used for variables not showing a normal distribution. The chi-square test was applied in the analysis of categorical variables.

Hierarchical multiple linear regression analyses were used to identify factors affecting insomnia severity (AIS total score), asthma control (ACT), and functionality (FAST total score) in asthma patients. Regression models were constructed stepwise according to theoretical and clinical priorities. In the first stage, demographic variables (age and body mass index) were included; in the second stage, a variable reflecting clinical disease control (ACT) was included; in the third stage, psychological symptom levels (DASS-21 depression, anxiety, and stress subscales) were included; in the fourth stage, a variable reflecting functionality or insomnia (depending on the model) was included; and in the final stage, social anhedonia (RSAS) was included in the model. The contribution of the variables added to the model at each stage to the explained variance was evaluated using R^2^ and ΔR^2^ values. Regression analyses for AIS, ACT, and FAST were performed as separate models, maintaining the same hierarchical approach for each dependent variable. The assumption of multicollinearity in regression analyses was examined using tolerance and VIF values; the independence of residuals was assessed using the Durbin–Watson test. The normality assumption of continuous variables was checked using visual and statistical methods. In all analyses, a two-tailed *p* < 0.05 value was considered statistically significant.

To determine the relationships between independent variables, Pearson correlation analyses were first performed. Mediator analyses were conducted to examine the indirect pathways between asthma control and social anhedonia and functioning, based on the relationships found to be significant in hierarchical regression analyses. Mediation analyses were performed using the PROCESS macro version 4.2 for SPSS (Model 4 and Model 6), indirect effects were calculated using 5000 bootstrap samples, and 95% confidence intervals were reported [26,27,28]. Indirect effects with confidence intervals not including zero were considered statistically significant [15].

This study was reported in accordance with the Strengthening the Reporting of Observational Studies in Epidemiology (STROBE) guidelines [29].

## 3. Results

A total of 153 asthma patients were included in the study. Of the participants, 102 were in the uncontrolled asthma group and 51 were in the controlled asthma group. No significant differences were found between the two groups in terms of age and gender distribution (*p* > 0.05). However, the body mass index (BMI) was significantly higher in the uncontrolled asthma group (31.72 ± 6.19 vs. 28.83 ± 4.90; *p* = 0.004).

The smoking burden (pack-years) was significantly higher in the uncontrolled asthma group (9.25 ± 14.9 vs. 4.33 ± 9.4; *p* = 0.034). The presence of atopic disease was more common in the uncontrolled asthma group (*p* = 0.023).

There is a significant difference between groups in terms of insomnia severity (*p* < 0.001). Moderate to severe insomnia is more common in the uncontrolled asthma group, while individuals without insomnia are more common in the controlled asthma group (Table 1).

Comparisons based on asthma control status are presented in Table 2. Insomnia severity is significantly higher in the uncontrolled asthma group (AIS: 9.43 ± 6.04 vs. 4.22 ± 4.83; *p* < 0.001). Similarly, depression, anxiety, and stress levels were also significantly higher in the uncontrolled asthma group (*p* < 0.001).

In contrast, no significant difference was found between the groups in terms of social anhedonia (RSAS) and overall functioning (FAST) total scores (*p* > 0.05). No significant differences were observed when examining the FAST subscales; only a borderline difference was observed in the leisure time subscale (*p* = 0.055).

Correlation analyses conducted as a preliminary analysis (Table S1) showed that asthma control was significantly and inversely related to insomnia severity and psychological symptoms. Insomnia severity showed a positive and significant relationship with social anhedonia and functioning. Social anhedonia also showed a significant correlation with functioning. These findings indicate that the statistical prerequisites for conducting subsequent regression and mediation analyses have been met.

The results of the hierarchical regression analysis conducted to determine the factors affecting insomnia severity (AIS total score) are presented in Table 3. In the first two stages, where demographic variables (age and BMI) were included in the model, the explained variance remained limited (R^2^ = 0.045). In the third stage, where asthma control (ACT) was added to the model, the variance explained increased significantly (ΔR^2^ = 0.279), and ACT was identified as a strong and independent predictor of insomnia severity (β = −0.683, *p* < 0.001).

The addition of psychological variables (depression, anxiety, and stress), functionality (FAST), and social anhedonia (RSAS) to the model did not result in a significant increase in explained variance. In the final model, ACT remained the sole independent predictor of insomnia severity (β = −0.451, *p* < 0.001) and the model explained 42.7% of the total variance (R^2^ = 0.427).

The results of the hierarchical regression analysis conducted to determine the factors affecting the level of functionality (FAST total score) are presented in Table 4. In the initial stages, where demographic variables (age and BMI) were included in the model, the variance explained remained limited (R^2^ = 0.031). The addition of asthma control (ACT) did not increase the explanatory power of the model, and ACT was not found to be an independent predictor (*p* > 0.05).

In the fourth stage, adding sleep disturbance severity (AIS) to the model increased the explained variance (ΔR^2^ = 0.029), and AIS was found to be significantly associated with functioning (β = 0.631, *p* = 0.035). However, the significance of AIS disappeared when psychological variables were added to the model.

In the final model, social anhedonia (RSAS) was identified as the only independent and significant predictor of functioning (β = 0.696, *p* = 0.033). Asthma control, insomnia, and psychological variables were not found to be independently significant in the final model. The final model explains 13.1% of the variance in functionality (R^2^ = 0.131).

**Figure 1:**
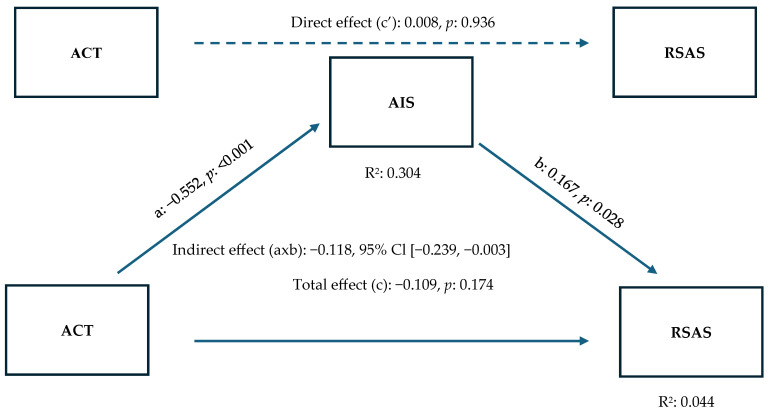
Mediation model (PROCESS Model 4) examining the direct and indirect effects of asthma control (ACT) on social anhedonia (RSAS). ACT: Asthma Control Test, AIS: Athens Insomnia Scale RSAS: Revised Social Anhedonia Scale. Unstandardized beta coefficients are reported. R^2^ values represent the variance explained.

Figure 1 shows a simple mediation model examining the relationship between asthma control (ACT) and social anhedonia (RSAS) mediated by insomnia severity (AIS). A strong and significant relationship was found between asthma control and insomnia (B = −0.6958, *p* < 0.001). Insomnia severity was found to be significantly related to social anhedonia (B = 0.1670, *p* = 0.0267). In contrast, the direct effect of asthma control on social anhedonia was not significant (B = 0.0075, *p* = 0.9362). The indirect effect was statistically significant (B = −0.1162, 95% CI [−0.2384, −0.0029]). These findings indicate that the relationship between asthma control and social anhedonia is largely mediated by insomnia severity.

**Figure 2:**
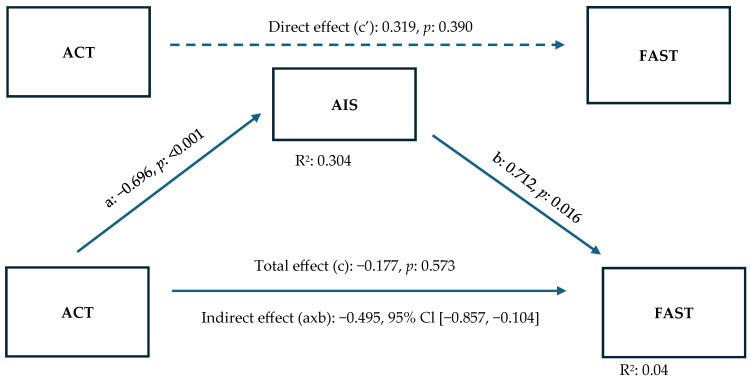
Mediation model (PROCESS Model 4) examining the direct and indirect effects of asthma control (ACT) on functioning assessment (FAST). ACT: Asthma Control Test, AIS: Athens Insomnia Scale FAST: Functioning Assessment Short Test. Unstandardized beta coefficients are reported. R^2^ values represent the variance explained.

Figure 2 shows the mediation model examining the relationship between asthma control (ACT) and functional status (FAST) through insomnia severity (AIS). A significant and strong relationship was found between asthma control and insomnia (B = −0.6958, *p* < 0.001). Insomnia severity was found to be significantly related to functionality (B = 0.7118, *p* = 0.0163). The direct effect of asthma control on functionality was not significant (B = 0.3186, *p* = 0.3896). However, the indirect effect was statistically significant (B = −0.4953, 95% CI [−0.8656, −0.1038]). This result suggests that the effect of asthma control on functionality is largely mediated by insomnia severity.

Figure S1 shows the serial mediation model examining the relationship between asthma control (ACT) and functional status (FAST) through insomnia severity (AIS) and social anhedonia (RSAS) (PROCESS Model 6). The serial mediation model was tested; however, the full sequential path was not statistically supported (Figure S1).

The results of the hierarchical regression analysis conducted to determine the variables affecting asthma control are presented in Table S2. In the final model, insomnia severity was found to be the strongest and independent predictor of asthma control. Anxiety level retained its significance after being added to the model. In contrast, depression, stress, functioning, and social anhedonia variables were found not to independently affect asthma control. The final model explains 42% of the variance in asthma control.

## 4. Discussion

The present study examined asthma control, insomnia severity, psychological symptoms, social anhedonia, and functional impairment within an integrated biopsychosocial framework. Several important findings emerged. First, uncontrolled asthma was associated with significantly greater insomnia severity and higher levels of depression, anxiety, and stress. Second, asthma control was the strongest independent predictor of insomnia severity. Third, although asthma control was not directly associated with social anhedonia or global functional impairment, insomnia significantly mediated these relationships. Importantly, the novelty of the present study lies not in demonstrating the coexistence of sleep disturbance and psychological symptoms in asthma, but in identifying insomnia as a potential intermediary process linking asthma control to psychosocial outcomes. These findings collectively suggest that sleep disturbance may represent a central pathway through which poor asthma control translates into psychosocial dysfunction.

Asthma is widely recognized as a chronic inflammatory airway disease with significant clinical and psychosocial burden [1,30]. However, the impact of the disease extends beyond airflow limitation and symptom frequency. Consistent with previous research, our uncontrolled asthma group exhibited significantly higher levels of depression, anxiety, and stress compared to controlled patients. High rates of emotional symptoms in asthmatic individuals are well documented [3,5], and meta-analytic evidence suggests a bidirectional relationship between asthma and depression [11]. Furthermore, psychological distress has been associated with poorer asthma control and increased symptom perception [4,31]. Our findings replicate this model and support the idea that asthma control is intertwined with emotional well-being.

Beyond emotional symptoms, sleep disturbance emerged as a key feature of uncontrolled asthma in our sample. Patients with uncontrolled asthma showed significantly higher insomnia severity, and asthma control independently predicted insomnia even after adjusting for demographic and psychological variables. This is consistent with epidemiological findings showing that insomnia is highly prevalent in asthma populations and closely related to disease control [6]. Longitudinal data further indicate a bidirectional association, whereby insomnia increases asthma risk and poorly controlled asthma exacerbates sleep disturbance [8]. Nocturnal respiratory symptoms, systemic inflammation, and heightened sympathetic activation may disrupt sleep continuity and promote hyperarousal [9,10]. Notably, in our hierarchical regression models, affective symptoms did not remain significant predictors of insomnia once asthma control was included, suggesting that sleep disturbance in asthma may be more directly linked to disease activity than to mood symptoms alone.

One of the most distinctive contributions of the present study is the examination of social anhedonia within the context of asthma. Social anhedonia, characterized by diminished pleasure derived from social interactions and reduced social motivation, is closely linked to reward-processing mechanisms [12,13]. In our findings, no significant difference in RSAS total scores was observed between the controlled and uncontrolled asthma groups at the direct comparison level. However, mediation analyses demonstrated that the relationship between asthma control and social anhedonia was not direct but was fully mediated by insomnia severity. In other words, asthma control did not exert a direct influence on social anhedonia; rather, poor asthma control was associated with increased insomnia, which in turn was associated with higher levels of social anhedonia. This indirect-only mediation pattern suggests that social anhedonia in asthma may emerge through intermediary mechanisms rather than as a direct consequence of disease severity.

The existing literature indicates that chronic illnesses are frequently associated with social withdrawal and reduced engagement in rewarding activities [2]. Experimental evidence from models of allergic lung inflammation suggests that peripheral inflammation may induce anhedonia-like behaviors through neuroimmune mechanisms [32]. Furthermore, inflammatory biomarkers associated with asthma severity have been linked to systemic immune activation [9], and large-scale cohort data indicate that insomnia may mediate inflammatory pathways related to mortality risk in asthma [10]. Our study, which identified functional impairment and social anhedonia as being associated with asthma, supports these findings.

When considered in light of these findings, our results suggest that the impact of poor asthma control on social anhedonia may operate through a sequential pathway. Poor asthma control may contribute to sleep disturbance, and persistent sleep disruption may subsequently influence reward-related processes. The absence of significant group differences in social anhedonia, coupled with the presence of significant indirect effects in mediation models, further supports the notion that social anhedonia may not represent a primary outcome of asthma severity, but rather a downstream consequence of sleep-related dysregulation. This interpretation is consistent with theoretical models proposing that chronic inflammation and sleep disturbance may interact to influence mesolimbic reward circuitry and motivational systems [9,32]. Overall, these findings are consistent with previously proposed biopsychosocial models suggesting that poor asthma control may contribute to insomnia, which may in turn increase vulnerability to social anhedonia and broader psychosocial dysfunction.

Functional impairment, assessed using the FAST, represents another clinically meaningful outcome. In our study, asthma control did not directly predict global functional impairment, and spirometric indices were not independently associated with psychosocial outcomes. This aligns with evidence suggesting that objective lung function does not fully capture patient-perceived impairment or daily functioning [1,3]. Insomnia initially predicted functional impairment; however, in the final model, social anhedonia emerged as the only independent predictor. This pattern suggests a cascading relationship in which sleep disturbance may influence functional outcomes partly through altered social motivation and reward processing. Prior research has demonstrated that social anhedonia is associated with occupational limitations, interpersonal dysfunction, and broader decrements in functioning [12,14]. Our findings extend this evidence to an asthma population and indicate that psychosocial functioning cannot be explained solely by respiratory symptom burden.

Importantly, the mediation analyses revealed indirect-only effects, indicating that insomnia may serve as a mechanistic bridge rather than a mere correlate. This pattern may reflect interconnected relationships between asthma control, sleep disturbance, and psychosocial functioning rather than direct effects of disease severity alone. Such a model is consistent with psychoneuroimmunological perspectives emphasizing interactions between systemic inflammation, sleep disruption, and central nervous system processes [2]. Within this framework, insomnia represents a potential therapeutic leverage point: addressing sleep disturbance may not only improve nocturnal symptoms but also mitigate downstream psychosocial consequences.

Overall, our findings suggest that asthma control should not be conceptualized exclusively in terms of respiratory parameters. The absence of direct associations between spirometric measures and psychosocial outcomes in our study underscores the complexity of asthma-related impairment. Instead, subjective disease control and sleep disturbance may play a more central role in shaping social engagement and daily functioning. By demonstrating that insomnia mediates the relationship between asthma control and both social anhedonia and functional impairment, this study highlights the importance of integrating sleep assessment into routine asthma management and supports a multidimensional understanding of disease burden.

These findings have important clinical implications. If insomnia represents a key mediator linking poor asthma control to psychosocial dysfunction, then targeted sleep interventions may improve not only sleep quality but also social engagement and functional outcomes. Cognitive behavioral therapy for insomnia (CBT-I) and integrated behavioral interventions may serve as adjunctive treatments in poorly controlled asthma populations.

Several limitations should be acknowledged in this study. Although mediation was tested in our study, causal inferences cannot be made due to the cross-sectional design. Longitudinal studies are needed to confirm the mediating role of insomnia over time. Additionally, inflammatory biomarkers were not directly measured; future studies incorporating cytokine profiling may further clarify the biological pathways underlying the observed associations. In addition, neurobehavioral or reward-processing measures were not assessed; therefore, interpretations regarding underlying motivational or reward-related mechanisms should be considered preliminary. The sample consisted of patients recruited from a single clinical setting, which may limit generalizability. Cultural, socioeconomic, and healthcare access factors could influence sleep patterns and psychosocial functioning in asthma populations. Despite these limitations, the study provides a clinically significant and theoretically integrative contribution by presenting new evidence that insomnia plays a central mediating role in linking asthma control and psychosocial dysfunction. Additionally, the study was not preregistered prior to data collection, which may limit methodological transparency.

## 5. Conclusions

In conclusion, this study identifies insomnia as a central mechanism linking poor asthma control to psychosocial dysfunction. Although uncontrolled asthma was associated with greater psychological distress, its effects on social anhedonia and functional impairment were primarily indirect and operated through insomnia severity. This mediation pattern underscores sleep disturbance as a key intermediary process rather than only a secondary consequence of emotional symptoms.

Clinically, these findings emphasize the importance of systematically assessing and treating insomnia in patients with poorly controlled asthma. Targeting sleep disturbance may not only improve nocturnal symptoms but also enhance social participation and overall functioning. Future longitudinal studies incorporating inflammatory and neurobehavioral markers are needed to better elucidate the mechanisms underlying the relationship between asthma control, sleep disturbance, and psychosocial outcomes.

## Figures and Tables

**Table 1:** Characteristics of participants (*n*: 153).

Variables	Uncontrolled Asthma (*n*: 102)	Controlled Asthma (*n*: 51)	*p* Value
**Age, years (mean ± SD)**	54.61 ± 13.89	53.43 ± 15.38	0.635
**BMI**	31.72 ± 6.19	28.83 ± 4.90	0.004
**Sex, *n* (%)**			0.836
Female	80 (78.4)	41 (80.4)	
Male	22 (21.6)	10 (19.6)	
**Marital status, *n* (%)**			0.50
Married	87 (85.3)	36 (70.6)	
Single	15 (14.7)	15 (29.4)	
Widowed/Divorced	0 (0)	0 (0)	
**Educational status**			0.049
Uneducated	10 (9.8)	1 (2)	
Elementary	66 (64.7)	27 (52.9)	
High School	16 (15.7)	15 (29.4)	
University	10 (9.8)	8 (15.7)	
**Employment status, *n* (%)**			0.923
Unemployed	20 (19.6)	9 (17.6)	
Employed	68 (66.7)	34 (66.7)	
Retired	14 (13.7)	8 (15.7)	
**Inhaler Medication**			0.156
ICS	30 (24.4)	9 (17.6)	
ICS + LABA	70 (68.6)	42 (82.4)	
ICS + LABA + LAMA	2 (2.0)	0 (0)	
**Smoking status, *n* (%)**			0.142
No	60 (58.8)	38 (74.5)	
Former smoker	42 (41.2)	13 (25.5)	
**Comorbidity, *n* (%)**			0.226
No	39 (38.2)	25 (49.0)	
Yes	63 (61.8)	26 (51.0)	
**Smoking (Pack-years)**	9.25 ± 14.9	4.33 ± 9.4	0.034
**Any Atopic Diseases**			0.023
Yes	48 (47.1)	14 (27.5)	
No	54 (52.9)	37 (72.5)	
**Asthma attack in the past 12 months**			0.110
Zero	50 (51.0)	36 (70.6)	
Once or twice	20 (19.6)	8 (15.7)	
Three or more than	30 (29.4)	7 (13.7)	
**Asthma attack requiring hospitalization in the last 12 months**			0.414
Yes	10 (9.8)	2 (3.9)	
No	101 (90.2)	49 (96.1)	
**Athens Insomnia Scale**			<0.001
Absence of Insomnia	29 (28.4)	41 (80.4)	
Mild Insomnia	31 (30.4)	6 (11.8)	
Moderate-severe Insomnia	42 (41.2)	4 (7.8)	

Variables with a normal distribution are presented as mean ± standard deviation, while non-normally distributed or ordinal variables are presented as median (25th–75th percentile). Categorical variables are expressed as counts and percentages. Group comparisons were performed using one-way ANOVA or Kruskal–Wallis test for continuous variables and chi-square test for categorical variables, as appropriate. Abbreviations: BMI: Body Mass Index, ICS: Inhaled corticosteroids, LABA: Long-acting β_2_-agonists, LAMA: Long-acting muscarinic antagonists.

**Table 2:** Relationships between clinical and psychological markers according to Asthma control.

	Uncontrolled Asthma (*n*: 102)	Controlled Asthma (*n*: 51)	*p*-Value
**FEV_1_ (%predicted)**	78.77 ± 16.81	82.76 ± 23.00	0.225
**FVC (%predicted)**	71.60 ± 15.36	73.92 ± 18.26	0.412
**AIS total score**	9.43 ± 6.04	4.22 ± 4.83	<0.001
**DASS-21**			
** -Depression**	12.61 ± 12.24	5.76 ± 7.43	<0.001
** -Anxiety**	15.53 ± 11.08	7.80 ± 7.22	<0.001
** -Stress**	14.98 ± 12.31	7.41 ± 8.40	<0.001
**RSAS**	18.0 ± 5.06	17.0 ± 4.23	0.227
**FAST**	20.43 ± 17.31	21.37 ± 21.73	0.772
** -Autonomy**	2.94 ± 4.08	3.22 ± 4.72	0.711
** -Occupational functioning**	4.70 ± 4.98	4.41 ± 5.31	0.745
** -Cognitive functioning**	4.62 ± 3.92	4.94 ± 4.75	0.655
** -Financial functioning**	1.49 ± 2.05	1.65 ± 2.39	0.674
** -Interpersonal relationships**	4.11 ± 5.31	5.27 ± 6.07	0.225
** -Leisure time**	2.58 ± 2.05	1.88 ± 2.17	0.055

Variables with a normal distribution are presented as mean ± standard deviation, while non-normally distributed. Group comparisons were performed using student’ *t* test or Man Whitney U for continuous variables and chi-square test for categorical variables, as appropriate. Abbreviations: AIS: Athens Insomnia Scale, FAST: Functioning Assessment Short Test; FEV_1_: forced expiratory volume in one second; FVC: forced vital capacity; DASS-21: Depression Anxiety Stress Scales-21, RSAS: Revised Social Anhedonia Scale.

**Table 3:** Hierarchical Regression Analysis Predicting Insomnia Severity (Athens Insomnia Scale).

Dependent Variable: Athens Insomnia Scale Total Score (AIS)					
Model	Variables	β	*p*	R^2^	ΔR^2^
**Model 1**	Age	0.062	0.076	0.021	0.021
**Model 2**	Age	0.036	0.328	0.045	0.024
	BMI	0.174	0.052		
**Model 3**	Age	0.052	0.093	0.324	0.279
	BMI	0.038	0.618		
	ACT	−0.683	<0.001		
**Model 4**	Age	0.057	0.054	0.416	0.092
	BMI	0.028	0.697		
	ACT	−0.436	<0.001		
	DASS-21—Depression	0.058	0.537		
	DASS-21—Anxiety	0.145	0.122		
	DASS-21—Stress	0.009	0.929		
**Model 5**	Age	0.054	0.073	0.420	0.005
	BMI	0.021	0.771		
	ACT	−0.448	<0.001		
	DASS-21—Depression	0.060	0.526		
	DASS-21—Anxiety	0.134	0.156		
	DASS-21—Stress	0.006	0.953		
	FAST	0.023	0.288		
**Model 6**	Age	0.058	0.052	0.427	0.007
	BMI	0.025	0.727		
	ACT	−0.451	<0.001		
	DASS-21—Depression	0.058	0.538		
	DASS-21—Anxiety	0.134	0.153		
	DASS-21—Stress	−0.005	0.955		
	FAST	0.018	0.417		
	RSAS	0.111	0.203		

ΔR^2^ *p* < 0.001. Abbreviations: ACT: Asthma Control Test, AIS: Athens Insomnia Scale, BMI: Body Mass Index, FAST: Functioning Assessment Short Test; DASS-21: Depression Anxiety Stress Scales-21, RSAS: Revised Social Anhedonia Scale.

**Table 4:** Hierarchical Regression Analysis Predicting Functional Impairment.

Dependent Variable: Functional Impairment (FAST Total Score)					
Model	Variables	β	*p*	R^2^	ΔR^2^
**Model 1**	Age	0.187	0.078	0.020	0.020
**Model 2**	Age	0.135	0.233	0.031	0.011
	BMI	0.352	0.200		
**Model 3**	Age	0.137	0.228	0.032	0.000
	BMI	0.335	0.235		
	ACT	−0.083	0.795		
**Model 4**	Age	0.104	0.359	0.060	0.029
	BMI	0.311	0.265		
	ACT	0.348	0.353		
	AIS	0.631	0.035		
**Model 5**	Age	0.139	0.222	0.103	0.043
	BMI	0.293	0.289		
	ACT	0.699	0.078		
	AIS	0.335	0.288		
	DASS-21—Depression	−0.087	0.808		
	DASS-21—Anxiety	0.429	0.231		
	DASS-21—Stress	0.124	0.735		
**Model 6**	Age	0.169	0.136	0.131	0.028
	BMI	0.312	0.254		
	ACT	0.632	0.107		
	AIS	0.254	0.417		
	DASS-21—Depression	−0.092	0.795		
	DASS-21—Anxiety	0.428	0.226		
	DASS-21—Stress	0.051	0.889		
	RSAS	0.696	0.033		

ΔR^2^ *p* < 0.001. Abbreviations: ACT: Asthma Control Test, AIS: Athens Insomnia Scale, BMI: Body Mass Index, FAST: Functioning Assessment Short Test; DASS-21: Depression Anxiety Stress Scales-21, RSAS: Revised Social Anhedonia Scale.

## Data Availability

The datasets generated and/or analyzed during the current study are not publicly available due to institutional policies and the presence of potentially identifiable patient information. However, anonymized data may be made available from the corresponding author upon reasonable request and with permission from the relevant ethics committee/institution.

## Supplementary Materials

The following supporting information can be downloaded at https://www.mdpi.com/article/10.3390/healthcare14111446/s1. Table S1. Pearson Correlations Between Asthma Control, Insomnia, Psychological Symptoms, Social Anhedonia, and Functional Impairment (*n* = 153). Table S2. Hierarchical Regression Analysis Predicting Asthma Control (Asthma Control Test Score). Figure S1: Serial mediation model (PROCESS Model 6) examining the direct and indirect effects of asthma control (ACT) on functioning assessment (FAST). ACT: Asthma Control Test, AIS: Athens Insomnia Scale FAST: Functioning Assessment Short Test, RSAS: Revised Social Anhedonia Scale.
