# Supplementary material for: Associations Between Asthma Control, Insomnia Severity, and Psychosocial Outcomes: A Cross-Sectional Mediation Analysis
Source: Healthcare (Basel). 2026 May 23;14(11):1446. doi: 10.3390/healthcare14111446 (PMC13257098; doi:10.3390/healthcare14111446)
Supplement: Supplementary file 1 [file healthcare-14-01446-s001.zip › healthcare-4221663-supplementary.pdf]

Table S1 shows the Pearson correlation coefficients between the study variables. Asthma control (ACT) was found to be significantly and inversely correlated with insomnia severity (AIS) ($r = -0.552$, $p < 0.01$), depression ($r = -0.495$, $p < 0.01$), anxiety ($r = -0.552$, $p < 0.01$), and stress ($r = -0.495$, $p < 0.01$). Insomnia severity was positively correlated with social anhedonia (RSAS) ($r = 0.210$, $p < 0.01$) and functionality (FAST) ($r = 0.187$, $p < 0.05$). Social anhedonia showed a significant correlation with functionality ($r = 0.217$, $p < 0.01$). In the sub-domain analysis of FAST, autonomy, interpersonal functionality, and leisure domains showed stronger relationships with psychological variables. These findings support the prerequisites for regression and mediation analyses.

Table S1. Pearson Correlations Between Asthma Control, Insomnia, Psychological Symptoms, Social Anhedonia, and Functional Impairment (n = 153)

**Variables**	1	2	3	4	5	6
**1. ACT score**	1					
**2. AIS (Insomnia)**	−0.552**	1				
**3. Depression (DASS-21)**	−0.495**	0.520**	1			
**4. Anxiety (DASS-21)**	−0.552**	0.549**	0.885**	1		
**5. Stress (DASS-21)**	−0.495**	0.511**	0.920**	0.898**	1	
**6. FAST**	−0.046	0.187*	0.213**	0.233**	0.220**	1
**7. RSAS**	−0.110	0.210**	0.275**	0.273**	0.298**	0.217**

Expanded correlations with FAST subdomains

**FAST Subdomains**	**AIS**	**Depression**	**Anxiety**	**Stress**	**ACT**	**RSAS**
**Autonomy**	0.235**	0.230**	0.265**	0.224**	−0.046	0.149
**Occupational**	0.002	0.075	0.079	0.065	−0.062	0.258**
**Cognitive**	0.123	0.131	0.152	0.146	0.015	0.249**
**Financial**	0.160*	0.239**	0.252**	0.237**	−0.026	0.086
**Interpersonal**	0.200*	0.214**	0.226**	0.240**	0.020	0.106
**Leisure time**	0.252**	0.177*	0.195*	0.181*	−0.225**	0.148

ACT: Asthma Control Test, AIS: Athens Insomnia Scale, FAST: Functioning Assessment Short Test; FEV1: forced expiratory volume in one second; FVC: forced vital capacity; DASS-21: Depression Anxiety Stress Scales-21, RSAS: Revised Social Anhedonia Scale

Pearson correlation coefficients are shown.

* $p < 0.05$, ** $p < 0.01$ (two-tailed).

Table S2 shows the results of the hierarchical regression analysis predicting asthma control (ACT). In the final model (Model 6), sleep disturbance severity (AIS) was found to be the strongest and independent predictor of asthma control ($\beta = -0.286$, $p < 0.001$). Anxiety level was also independently significant ($\beta = -0.172$, $p = 0.021$). Depression, stress, functionality (FAST), and social anhedonia (RSAS) did not independently predict asthma control. The total variance explained by the model is 42% ($R^2 = 0.422$). These results support the strong association between asthma control and sleep disturbance and anxiety in particular.

Table S2. Hierarchical Regression Analysis Predicting Asthma Control (Asthma Control Test Score)

Dependent variable: Asthma Control Test score (ACT)

**Model**	**Variables**	**β**	**p**	**R^2**	**ΔR^2**
**Model 1**	Age	−0.005	0.850	0.00	0.00
**Model 2**	Age	0.024	0.407	0.051	0.051
	BMI	−0.198	0.005		
**Model 3**	Age	0.039	0.110	0.328	0.277
	BMI	−0.124	0.041		
	AIS	−0.427	<0.001		
**Model 4**	Age	0.023	0.328	0.407	0.079
	BMI	−0.102	0.079		
	AIS	−0.278	<0001		
	DASS-21- Depression	−0.002	0.979		
	DASS-21- Anxiety	−0.164	0.028		
	DASS-21- Stress	0.008	0.917		
**Model 5**	Age	0.019	0.435	0.420	0.013
	BMI	−0.108	0.060		
	AIS	−0.282	<0.001		
	DASS-21- Depression	0.001	0.992		
	DASS-21- Anxiety	−0.173	0.020		
	DASS-21- Stress	0.004	0.958		
	FAST	0.031	0.078		
**Model 6**	Age	0.021	0.389	0.422	0.002
	BMI	−0.106	0.066		
	AIS	−0.286	<0.001		
	DASS-21- Depression	0.000	0.998		
	DASS-21- Anxiety	−0.172	0.021		
	DASS-21- Stress	−0.001	0.995		
	FAST	0.028	0.107		
	RSAS	0.046	0.511		

* ΔR^2 p < 0,001

Abbreviations: ACT: Asthma Control Test, AIS: Athens Insomnia Scale, BMI: Body Mass Index, FAST: Functioning Assessment Short Test; FEV1: forced expiratory volume in one second; FVC: forced vital capacity; DASS-21: Depression Anxiety Stress Scales-21, RSAS: Revised Social Anhedonia Scale

Figure S1 illustrates a series mediation model (PROCESS Model 6) in which the relationship between asthma control (ACT) and level of functionality (FAST) is examined via insomnia severity (AIS) and social anhedonia (RSAS). A significant association was found between asthma control and insomnia (B = -0.6958, p < 0.001). Insomnia severity was significantly associated with both social anhedonia (B = 0.1670, p = 0.0267) and functionality (B = 0.5905, p = 0.0461). Social anhedonia was also independently associated with functionality (B = 0.7263, p = 0.0228). However, the complete series indirect pathway (ACT → AIS → RSAS → FAST) was not statistically significant. Therefore, the model should be considered exploratory in nature.

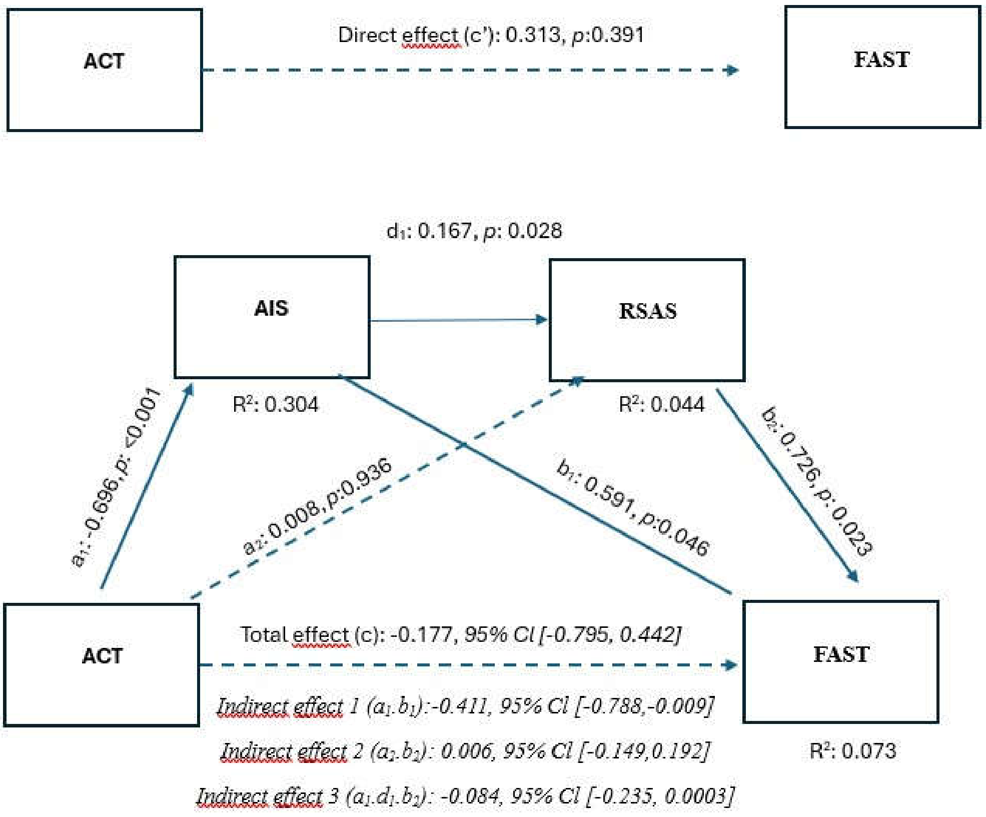

Unstandardized beta coefficients are reported. R^2 values represent the variance explained.

Figure S1: Serial mediation model (PROCESS Model 6) examining the direct and indirect effects of asthma control (ACT) on functioning assessment (FAST). **ACT:** Asthma Control Test, **AIS:** Athens Insomnia Scale **FAST:** Functioning Assessment Short Test, **RSAS:** Revised Social Anhedonia Scale
